# A call for *BMC Research Notes *contributions promoting best practice in data standardization, sharing and publication

**DOI:** 10.1186/1756-0500-3-235

**Published:** 2010-09-02

**Authors:** Iain Hrynaszkiewicz

**Affiliations:** 1 Associate Journal Publisher, BioMed Central, 236 Gray's Inn Road, London WC1X 8HB UK

## Abstract

*BMC Research Notes *aims to ensure that data files underlying published articles are made available in standard, reusable formats, and the journal is calling for contributions from the scientific community to achieve this goal. Educational Data Notes included in this special series should describe a domain-specific data standard and provide an example data set with the article, or a link to data that are permanently hosted elsewhere. The contributions should also provide some evidence of the data standard's application and preparation guidance that could be used by others wishing to conduct similar experiments. The journal is also keen to receive contributions on broader aspects of scientific data sharing, archiving, and open data.

## Editorial

It has always been a key objective of *BMC Research Notes *to ensure that data files associated with peer-reviewed articles will, wherever possible, be published in standard, reusable formats and be exposed to ensure that they are searchable and easily harvested for reuse [1]. The article published today in *BMC Research Notes *by Vickers and Cronin [2] is an excellent example of clean, well-annotated and re-usable data that have been made freely available by this innovative publication policy.

Across the spectrum of biomedical research myriad domain-specific data file standards exist, and to promote data sharing - and publication - we aim to provide 'Additional data file' preparation guidelines, to complement BioMed Central's current figure preparation guidelines. These guidelines should serve as a useful resource to researchers wanting to prepare or share data and will include links to relevant external sources, including published examples, as well as original information and guidance.

Of course, in certain fields widely agreed and accepted data standards already exist. Our preparation guidelines will, for example, recommend that authors reporting microarray experiments prepare their data according to the Minimum Information About a Microarray Experiment MIAME guidelines [3], and will recommend using the spreadsheet-based MAGE-TAB format [4].

Different disciplines, however, have embraced the possibilities of data sharing and open data to differing extents, and it can take the leadership of a small number of individuals to develop and promote their standard to secure widespread adoption, and enable interoperability of scientific data (this was one of the motivations for BioMed Central's Open Data Award [5]). In other cases a standard of data collection and preparation might be well known amongst circles of experts but perhaps unknown to researchers in different or even related fields. But with few journals considering data-driven articles and apparent inconsistencies in incentives and rewards for data publication, the availability of definitive and freely-available examples of re-usable, standardized data across the life sciences is patchy at best.

By publishing Data Notes (often called "data papers" by other publishers), authors in *BMC Research Notes *can publish peer-reviewed articles that briefly describe a biomedical data set or database, with the data being readily accessible and attributed to a source. So far we have only attracted small numbers of these articles. The majority of authors have so far used the journal for another - equally important - reason, that of completing the scientific record by publishing sound small-scale, confirmatory or negative studies that might otherwise go unpublished.

The *BMC Research Notes *editorial team believe that these facts, combined with the shift towards data-intensive science and the inevitable need for multi-disciplinary projects [6], warrant the publication of a series of educational articles that promote best practice in data sharing across biology and medicine. We are therefore seeking authors to contribute an article to the journal that describes a data standard and how a reference data set has been prepared in line with that standard, preferably with the associated data set as an additional file to serve as a concrete example if possible. Given the importance of promoting the sharing of reusable data for the future of scholarly communication we are treating contributions to this series as commissioned, educational content, and will waive the journal's article processing charge.

Articles should follow a similar format to that described in the Data Note instructions for authors [7], but there are a small number of additional considerations for contributors to this special, educational series that is presenting data along with guidance and best practice for data sharing:

1. Evidence of use

Authors must provide evidence of some pre-existing use of the data standard described in their article, and a short justification of what value this example and description of their standard will add to the literature.

2. Universally available, re-usable and standardized data

The data set must be freely and permanently available with no restrictions on access. It can either be included with the published article (additional files are unlimited in number within reason but should be no greater than 20 Mb each) or publicly available but hosted elsewhere. Data hosted elsewhere must be available in perpetuity with permanence guaranteed -repositories that provide a digital object identifier (DOI) or equivalent for data, such as Dryad [8], are available and are growing in number. Of course, the data need to be clean, and each variable annotated to the extent that another researcher could independently repeat previous analyses or conduct new analyses.

The use of open standards, such as XML and PNG, has been recommended for open data [9], although widely used closed file formats such as Microsoft Excel are often useful [10]. Therefore, if both open/raw formats and a widely-used closed format are available, such as in the article by Vickers, [2] we recommend they be included. In any case we recommend that file formats be as general as possible.

3. Preparation guidance

Authors should include brief information on how their data set was prepared in line with their standard. This might seem elementary to the authors but could be valuable to researchers in other disciplines.

3. Novel contribution

By being novel we do not, in the traditional journal sense, mean the articles should present novel findings. However, we do mean to reinforce that this series of articles does not intend to reinvent the wheel. If a very widely documented and supported standard already exists, such as MAGE-TAB, then another example of this format and standard might not contribute something new to the data-sharing literature. Such standards however, will be recognised - and linked to - in the catalogue of standards we will refer to in the 'Additional data file' preparation guidelines that it is our intention to create.

As well as articles promoting and demonstrating specific data standards, we are also keen to receive contributions on broader aspects of scientific data sharing, archiving, and open data. For more information and to contribute please contact the author or the *BMC Research Notes *editorial office researchnotes@biomedcentral.com with a pre-submission enquiry.

## List of abbreviations

DOI: digital object identifier; MAGE-TAB: MicroArray Gene Expression Tabular; Mb: megabyte; MIAME: Minimum information about a microarray experiment; PNG: Portable Network Graphics; XML: Extensible Markup Language

## Competing interests

IH is employed by BioMed Central and receives a fixed salary, and is a supporter of data sharing and release from all types of scientific research.
